# Transfusion transmitted infections among male blood donors of White Nile State, Sudan: Screening of the current seroprevalence and distribution

**DOI:** 10.1186/s13104-020-05333-6

**Published:** 2020-11-30

**Authors:** Elnaim Bushra Ahmed, Areej Ahmed Essa, Babiker Saad Almugadam, Qawaeid Mohamed Ahmed, Mofeeda Mohammed Hussein

**Affiliations:** 1 Department of Microbiology, Faculty of Medical Laboratory Sciences, University of El Imam El Mahdi, Kosti, Sudan; 2 Department of Medical Laboratory Investigations, Kosti Police Hospital, Kosti, Sudan; 3 Deparment of Microecology, College of Basic Medical Sciences, Dalian Medical University, Dalian, China

**Keywords:** HIV, Blood donors, HBV, HCV, *T. palladium*, TTIs

## Abstract

**Objective:**

Our study planned to investigate the current positivity rate and distribution of the serologic markers of TTIs among male blood donors of the White Nile state, Sudan.

**Results:**

The overall reported seropositive cases of TTIs was 15.91%, and percentages of anti-Human immunodeficiency virus 1/2 (anti-HIV1/2), Hepatitis B virus surface antigen (HBVsAg), anti-Hepatitis C virus (anti-HCV), and anti-*Treponema palladium* (anti-*T. palladium*) were 2.61%, 5.57%, 1.40%, and 5.72%, respectively. Out of 10897 donors examined, 0.59% had a serological sign of multiple infections. Furthermore, the odds of testing positive for TTIs were higher in the 28–37 age group (OR: 2.620, 95% CI: 2.324–2.955) and lower in the 38–47 age group (OR: 0.671, 95% CI: 0.567–0.794) compared to individuals of 18–27 years old. Likewise, it is more in individuals of Kosti (OR: 1.122, 95% CI: 0.987–1.277) and Rabak (OR: 1.354, 95% CI: 1.188-1.543) localities compared to Al Douiem locality. Anti-HIV/anti-*T. palladium* (27.70%) and anti-HIV/HBVsAg (23.07%) were the most frequently detected serologic markers of co-infections, *P *= 0.002.

## Introduction

Transfusion-transmitted infections (TTIs) are a heterogeneous group of infectious diseases, which constitute major health challenges. Collectively, the Human immunodeficiency virus (HIV), Hepatitis B virus (HBV), Hepatitis C virus (HCV), and *T. palladium* infections represent the most common TTIs globally [1–5]. Moreover, HIV, HBV, and *T. palladium* are not only acquired through the infected blood but also can transmit by sexual contact, which make them easily and rapidly spread [6]. Formerly, to evade the probability of moving ailments from non-diagnosed cases to blood receivers, the World Health Organization (WHO) issued a statement of testing the blood donors before transfusion at least to HIV, HBV, HCV, and *T. palladium*, which are causes of AIDS, Hepatitis B, Hepatitis C, and Syphilis, respectively [7, 8].

Regardless of the availability of the advanced and rapid diagnostic tests globally, the epidemiology of these illnesses is poorly studied, and few data existed concerning TTIs in Sub-Saharan Africa [9]. Sudan classified as a country with a high HBV and HCV infections endemicity [10–12]. Limited studies on HCV infection in Sudan revealed a low (2.74%) seroprevalence. Although there was a continuous increase in the incidence of HIV infected individuals, the frequency of illness is unknown and the previous reported seroprevalence of HIV and HBV infections had ranged from 0 to 18.3% and 5.1 to 26.81%, respectively [10].

Investigating the TTIs can provide data about the magnitude of unnoticeable infected individuals in the community, which is helpful in diagnosis and therapy of such diseases, and in articulating health policies and protocols. Therefore, our study aimed to investigate the prevalence and distribution of serologic markers of TTIs, in particular, anti-HIV1/2, HBV surface antigen (HBVsAg), anti-HCV, and anti-*T. palladium* among volunteer male blood donors of White Nile state, Sudan.

## Main text

### Materials and methods

This was a single year retrospective epidemiological study conducted in the White Nile state of Sudan to gather the data of male volunteer blood donors out January 1, 2017 to December 31, 2017. Blood banks of Kosti, Al Douiem, and Rabak Teaching Hospitals have been selected as these are major hospitals in the state and situated in the most populated localities (Kosti, Al Douiem and Rabak, respectively), which were represented around 53.32% (940123) of the total state population according to the census of 2008.

The study population composed of male blood donors regardless of occupation, education level, and ethnic groups. The selection of volunteer blood donors based on a pre-set of inclusion criteria, and the main inclusion criteria were age between 18 and 65 years, weights over 45 kg, and free of illnesses as determined by past medical history, clinical examination, and laboratory investigations. While those did not fit these criteria or have a history of recent blood donation before less than 6 months were excluded. The data concerning the findings of blood bank investigations and the socio-demographic characteristics of blood donors were compiled from blood banks records. The gathered information includes age, locality, and blood banks investigations outcome for anti-HIV1/2, HBVsAg, anti-HCV, and anti-*T. palladium* tests*.*

The laboratory examination of TTIs was based on serologic tests. Briefly, a 5 ml of venous blood was collected from every donor in a sterile blood container without anti-coagulant and left at room temperature for few minutes to form a clot. Afterward, each sample had centrifuged at 3000 rpm for 10 min to obtain serum that immediately tested for TTIs by rapid Immunochromatographic test devices (Acon, USA) according to manufacturer instructions. The positive tests were indicated by the presence of red color in test and control lines.

The gathered data was analyzed by IBM SPSS software version 21. The prevalence of the serologic markers of TTIs, co-infections, anti-HIV1/2, HBVsAg, anti-HCV, and anti-*T. palladium*  were expressed as numbers and percentages. Pearson Chi-Squired and Fishers Exact tests were used to assess the statistical difference among the gathered data. The association between the serologic markers of TTIs or co-infections and their influencing factors were also evaluated using binary logistic regression. A *P* value of <0.05 was considered significant.

### Results

The data of 10,897 male blood donors were retrieved from blood bank records, of them, the information of 4152 (38.10%), 3414 (31.30%), and 3331(30.60%) donors have gathered from Kosti, Rabak, and Al Douiem locality, correspondingly. Furthermore, the majority of individuals 4183 (38.39%) were aged 28-37 years as well as most of them 4152 (38.10%) were Kosti locality residents, Table 1.

**Table 1:** Distribution of the study population among areas and age groups, and seroprevalence (Positivity rate) of the serologic markers of transfusion-transmitted infections (TTIs)

Variable	Blood donors: N (%)	Prevalence: N (%)					
		Anti-HIV1/2	HBVsAg	Anti-HCV	*Anti*-*T. palladium*	MI	Total of TTIs
Overall	10897	285 (2.61)	607 (5.57)	153 (1.40)	624 (5.72)	65 (0.59)	1734 (15.91)
Age							
18–27 years	3950 (26.25)	32 (0.81)	157 (3.97)	42 (1.06)	197 (4.98)	25 (0.63)	453 (11.46)
28–37 years	4183 (38.39)	225 (5.37)	356 (8.51)	103 (2.46)	355 (8.48)	21 (0.50)	1060 (25.34)
38–47 years	2764 (25.36)	28 (1.01)	94 (3.40)	8 (0.28)	72 (2.60)	19 (0.68)	221 (7.99)
*P* value	< 0.001	< 0.001	< 0.001	< 0.001	< 0.001	0.576	< 0.001
Residence							
Al Douiem	3331 (30.60)	47 (1.41)	179 (5.37)	62 (1.86)	169 (5.07)	12 (0.36)	469 (14.07)
Kosti	4152 (38.10)	164 (3.94)	211 (5.08)	28 (0.67)	215 (5.17)	27 (0.65)	645 (15.53)
Rabak	3414 (31.30)	74 (2.16)	217 (6.35)	63 (1.84)	240 (7.02)	26 (0.76)	620 (18.16)
*P* value	< 0.001	< 0.001	0.047	< 0.001	< 0.001	0.086	< 0.001

*TTIs* Transfusion-transmitted infections, *N* Number, *HIV* Human immunodeficiency virus, *HBVsAg* Hepatitis B virus surface antigen, *HCV* Hepatitis C virus, *MI* Multiple infections

Statistical differences were evaluated using Pearson Chi-Squared test

Out of total donors, 1734 (15.91%) were tested positive for TTIs. Likewise, 1669 (15.31%) had a serological marker of a single type of TTIs. Moreover, there was a statistically significant difference in the occurrence of overall serologic markers of TTIs among age groups and geographic areas. In particular, the donors of age group 28–37 years (25.34%) had a higher frequency of the serologic markers of TTIs compared with 18–27 (11.46%) and 38–47 (7.99%) age groups. The blood donors of Rabak locality had shown a higher percentage of seropositivity for TTIs (18.16%) compared with other localities, Table 1. Furthermore, the odds of testing positive for TTIs were statistically much higher in 28–37 age group (OR: 2.620, 95% CI: 2.324–2.955), while, much lower in 38–47 age group (OR: 0.671, 95% CI: 0.567–0.794) compared to individuals of 18–27 years old. Likewise, it is more in individuals of Kosti (OR: 1.122, 95% CI: 0.987–1.277) and Rabak (OR: 1.354, 95% CI: 1.188–1.543) localities compared to Al Douiem locality, Table 2.

**Table 2:** Logistic regression analysis for the serologic markers of TTIs and MI

Variable	TTIs		MI among TTIs positive cases	
	OR (95% CI)	*P* value	OR (95% CI)	*P* value
Age				
18–27 years	1	–	1	–
28–37 years	2.620 (2.324–2.955)	< 0.001	0.346 (0.192–0.625)	< 0.001
38–47 years	0.671 (0.567–0.794)	< 0.001	1.610 (0.867–2.992)	0.132
Residence				
Al Douiem	1	–	1	–
Kosti	1.122 (0.987–1.277)	0.079	2.100 (1.053–4.189)	0.031
Rabak	1.354 (1.188–1.543)	< 0.001	1.893 (0.946–3.790)	0.072

*TTIs* Transfusion-transmitted infections, *MI* Multiple infections, *OR* Odd ratio, *CI* Confidence interval

The overall stated prevalence of anti-HIV1/2, HBVsAg, anti-HCV, and anti-*T. palladium* were 2.61%, 5.57%, 1.40%, 5.72%, respectively. Notably, anti- *T. palladium* and HBVsAg are the major detected markers of TTIs, with the highest frequency among the blood donors of Rabak locality and individuals of 28–37 years old. Furthermore, there was a statistically significant difference in the seroprevalence of these markers among age and geographic areas. Particularly, blood donors of 28-37 years old had a higher percentage of anti-HIV1/2 (5.37%), anti-HCV (2.46%), HBVsAg (8.51%), and anti-*T. palladium* (8.48%) compared to other age groups. Regionally, the donors of Rabak locality had a high rate of HBVsAg (6.35%) and anti-*T. palladium* (7.02%), whereas, the donors from Kosti locality had shown a higher frequency of anti-HIV (3.94%) , Table 1.

Notably, the prevalence of co-infections markers was 65 (0.59%). Moreover, there was no statistical difference in the occurrence of the serologic markers of multiple infections (MI) among age groups and study areas, *P *= 0.576 and 0.086, respectively, Table 1.

Additional file 1: Figure S1a-c displayed the probability of the serologic markers of MI among TTIs seropositive cases. Out of the total of TTIs seropositive cases, 3.74% have shown the serologic indication of co-infections, Additional file 1: Figure S1a. Likewise, the probability of the serologic markers of MI was a significantly dissimilar among TTIs positive cases in blood donors of different age groups, and represented 5.51%, 1.98%, and 8.59% for 18–27, 28–37, 38–47 years age groups, respectively, Additional file 1: Figure S1b. The regression analysis has revealed that the individuals of 28–37 years age (OR: 0.346, 95% CI: 0.192–0.625) were statistically less likely to have the serologic sign of MI compared to individuals of 18–27 years age. Moreover, the odd of testing positive for the serologic markers of MI were much greater among the people of Kosti (OR: 2.100, 95% CI 1.053–4.189) compared to Al Douiem locality, Table 2.

Table 3 summarizes the co-occurrence of co-infections markers among the MI positive cases. Comparatively, anti-HIV/anti-HCV and anti-HCV/anti-*T. palladium* were more common in individuals of 38–47 years old and Rabak locality; however, it is not significant except for anti-HIV/anti-HCV among age groups. Furthermore, there was a statistically significant difference concerning the co-occurrence of the serologic markers of MI in 18–27 and 28–37 years age groups as well as in Rabak and Al Douiem localities. Notably, anti-HIV/anti-*T. palladium*  represnts 27.70% of overall markers of MI (*P* = 0.002). A 36% of  anti-HIV/anti-*T. palladium*  was detected in 18–27 years age group, whereas 33.33% of anti-HIV/HBVsAg had seen in 28–37 years age group and 36.84% of HBVsAg/anti-*T. palladium* reported in individuals of ages ranged from 38–47 years old. Regionally, 29.63% of anti-HIV/HBVsAg was found in Kosti locality, whereas 34.62% of  anti-HIV/anti-*T. palladium* detected in the individuals of Rabak locality. Moreover, 41.67% of HBVsAg/anti-*T. palladium*  was reported in Al Douiem locality, Table 3.

**Table 3:** Co-occurrence of TTIs serologic markers among the MI positive cases

Variable	Total of MI	Co-occurrence: % (N)						*P* value
		anti-HIV/HBVsAg	anti-HIV/anti-HCV	anti-HIV/anti-TP	HBVsAg/anti-HCV	HBVsAg/anti-TP	anti-HCV/anti-TP	
Overall	65	23.07 (15)	16.92 (11)	27.70 (18)	4.61 (3)	20 (13)	7.70 (5)	0.002^a^
Age								
18–27 years	25	24 (6)	0 (0)	36 (9)	4 (1)	28 (7)	8 (2)	0.001
28–37 years	21	33.33 (7)	19.05 (4)	28.57 (6)	0 (0)	14.29 (3)	4.76 (1)	0.014
38–47 years	19	10.52 (2)	36.84 (7)	15.80 (3)	10.52 (2)	15.80 (3)	10.52 (2)	0.316
*P* value	0.567	0.139	0.008	0.122	0.679	0.196	1.000	
Residence								
Al Douiem	12	25 (3)	8.33 (1)	25 (3)	0 (0)	41.67 (5)	0 (0)	0.025
Kosti	27	29.63 (8)	14.82 (4)	22.22 (6)	11.11 (3)	14.82 (4)	7.40 (2)	0.320
Rabak	26	15.38 (4)	23.08 (6)	34.62 (9)	0 (0)	15.38 (4)	11.54 (3)	0.017
*P* value	0.006	0.208	0.100	0.122	0.036	1.000	0.251	

*MI* Multiple infections, *N* Number, *HIV* Human immunodeficiency virus, *HBVsAg* Hepatitis B virus surface antigen, *HCV* Hepatitis C virus, *TP T. palladium*

Pearson Chi squared^a^ and Fishers Exact test have involved in the Statistical analysis

### Discussion

Every year, transfusions therapies of blood and it’s constituents save millions of lives and decrease the morbidity of illnesses globally [13]. Nevertheless, to avoid its accompanied biohazards and harm, the physicians and health care practitioners should ensure the transfusion of safe blood to recipients. In this regard, the screening of blood donors for TTIs is a helpful in prevention against the life threatening infections. The reliable screening approach for TTIs was nucleic acid amplification methods, which enable the early discovery of infectious agents. Western blot, indirect fluorescent antibody, recombinantimmunoblot, and enzyme-linked immunosorbent assays were also major reliable screening methods. Unfortunately, these methods are not available in resource-limited settings such as the major areas of developing countries where rapid diagnostic tests (Screening and non-confirmatory) have been widely adopted [14]. In this study, the majority (38.39%) of blood donors were age range 28–37 years, which was analogous to the prvious studies outcomes [15–17]. On the other hand, the percentage of overall donors tested psotive for TTIs (15.91%) or, in particular, anti-HIV1/2 (2.61%), anti-HCV (1.40%), HBVsAg (5.57%), anti-*T. palladium* (5.72%), and MI (0.59%) are much higher in this study compared with the findings of many previous studies [3, 8, 15, 18]. In contrast, there are lowered compared to Bisetegen FS et al., Study [9]. The probable reasons for the dissimilarity between these studies may attribute to the difference in the study area and population, sample size, socioeconomic status and cultural habits of study communities, and diagnostic methods, which are known elements affecting the occurrence and distribution of various illnesses. Moreover, the lack of health survey and education programs in our study area could also be responsible.

Notably, Rabak residents and the individuals of ages range 28–37 years comprised the majority of TTIs positive cases. Although these outcomes have also strengthened by the findings of multivariate logistic regression, Pearson Chi -Squared analysis found that the higher prevalence among the Rabak residents was only occurred by the significant greater frequency of HBVsAg, and anti-*T. palladium*. Formerly, Rebouças KA et al., study has documented similar probability of TTIs among age groups [18], which could be related to the dissimilarity in the genetic constitution, immunity of individuals, and the magnitude of risk factors that may affect the individual’s susceptibility to TTIs. Likewise, it has known that the period of living in the infected community increases the likelihood of acquiring illnesses. On the other hand, Rabak is formerly suffering from more poverty and lack of health services compared to Kosti and Al Douiem locality, which are factors that extremely affect infection rates and distribution. Our findings have drawn attention to look for the real reason of the higher prevalence in this age group and area.

Concerning the co-occurrence of the serologic markers of TTIs among the MI positive cases, the probability of having anti-HIV/anti-*T. palladium* (27.70%) was statistically much higher among the individuals of White Nile state. Furthermore, we also found that the possibility and co-occurrence of the serologic markers of MI is extremely affected by age and region. This could increase our understanding regarding the relation underlying TTIs interactions, which need further deep studies. Moreover, it is also a necessary for the formulation and application of public health strategies and polices.

### Conclusions

Our study  screened the rate of the serologic markers of TTIs in the White Nile state and it’s distribution among study areas and age groups, and indicated the need for deep studies to a better understanding of the epidemiology and risk factors of TTIs. Likewise, it is underscored the need for public health surveillance, therapeutic, and preventive programs.

## Limitation

The study limitations include a low sample size, use of rapid diagnostic test, and lacks of several socio-demographic features of the blood donor’s such as occupation, blood group and rhesus, and education level.

## Supplementary information

**Additional file 1: Figure S1.** Rate of the serologic markers of multiple infections (MI) among TTIs positive cases. **a** overall MI. **b** and **c** show the rate of MI among age groups and localities, respectively. Pearson Chi -Squared test assessed the difference between groups. *P* value was < 0.001 (Age groups) and 0.088 (localities).

## Data Availability

The datasets analysed during the current study are available from the corresponding author on reasonable request.

## Abbreviations

HBV: Hepatitis B virus
HBVsAg: Hepatitis B virus surface antigen
HCV: Hepatitis C virus
HIV: Human immunodeficiency virus
MI: Multiple infections
TTIs: Transfusion-transmitted infections
WHO: World Health Organization

## References

[1] Keshvari M, Sharafi H, Alavian SM, Mehrabadi H, Zolfaghari S (2015). Prevalence and trends of transfusion-transmitted infections among blood donors in Tehran, Iran from 2008 to 2013. Transfus Apher Sci.

[2] Tagny CT, Kouao MD, Touré H, Gargouri J, Fazul AS, Ouattara S (2012). Transfusion safety in francophone African countries: an analysis of strategies for the medical selection of blood donors. Transfusion.

[3] Song Y, Bian Y, Petzold M, Ung CO (2014). Prevalence and trend of major transfusion-transmissible infections among blood donors in Western China, 2005 through 2010. PLoS One..

[4] Gharehbaghian A (2011). An estimate of transfusion-transmitted infection prevalence in general populations. Hepat Mon.

[5] Tessema B, Yismaw G, Kassu A, Amsalu A, Mulu A, Emmrich F (2010). Seroprevalence of HIV, HBV, HCV and syphilis infections among blood donors at Gondar University Teaching Hospital, Northwest Ethiopia: declining trends over a period of five years. BMC Infect Dis.

[6] Soares CC, Georg I, Lampe E, Lewis L, Morgado MG, Nicol AF (2014). HIV-1, HBV, HCV, HTLV, HPV-16/18, and Treponema pallidum infections in a sample of Brazilian men who have sex with men. PLoS ONE.

[7] World Health Organization. Screening donated blood for transfusion-transmissible infections: recommendations. World Health Organization. 2009. https://apps.who.int/iris/handle/10665/44202. Accessed 10 Feb 2020. 23741773

[8] Yang S, Jiao D, Liu C, Lv M, Li S, Chen Z (2016). Seroprevalence of human immunodeficiency virus, hepatitis B and C viruses, and Treponema pallidum infections among blood donors at Shiyan, Central China. BMC Infect Dis.

[9] Bisetegen FS, Bekele FB, Ageru TA, Wada FW (2016). Transfusion-Transmissible Infections among Voluntary Blood Donors at Wolaita Sodo University Teaching Referral Hospital, South Ethiopia. Can J Infect Dis Med Microbiol.

[10] Badawi MM, Atif MS, Mustafa YY (2018). Systematic review and meta-analysis of HIV, HBV and HCV infection prevalence in Sudan. Virol J.

[11] Mudawi HM, Smith HM, Rahoud SA, Fletcher IA, Saeed OK, Fedail SS (2007). Prevalence of hepatitis B virus infection in the Gezira state of central Sudan. Saudi J Gastroenterol.

[12] McCarthy MC, Hyams KC, el-Tigani el-Hag A, el-Dabi MA, el-Sadig el-Tayeb M, Khalid IO, et al. HIV-1 and hepatitis B transmission in Sudan. AIDS. 1989;3(11):725-9. 10.1097/00002030-198911000-00006. 10.1097/00002030-198911000-00006 2515878

[13] Sharma S, Sharma P, Tyler LN (2011). Transfusion of blood and blood products: indications and complications. Am Fam Physician.

[14] Mbanya D (2013). Use of quality rapid diagnostic testing for safe blood transfusion in resource-limited settings. Clin Microbiol Infect.

[15] Siraj N, Achila OO, Issac J, Menghisteab E, Hailemariam M, Hagos S (2018). Seroprevalence of transfusion-transmissible infections among blood donors at National Blood Transfusion Service, Eritrea: a seven-year retrospective study. BMC Infect Dis.

[16] Mohammed Y, Bekele A (2016). Seroprevalence of transfusion transmitted infection among blood donors at Jijiga blood bank, Eastern Ethiopia: retrospective 4 years study. BMC Res Notes.

[17] Nagalo BM, Bisseye C, Sanou M, Kienou K, Nebié YK, Kiba A (2012). Seroprevalence and incidence of transfusion-transmitted infectious diseases among blood donors from regional blood transfusion centres in Burkina Faso West Africa. Trop Med Int Health.

[18] Rebouças KAAF, Narici FM, Santos Junior MN, Neres NSM, Oliveira MV, Souza CL (2019). Seroprevalence of transfusion-transmissible infectious diseases at a hemotherapy service located in southwest Bahia Brazil. Hematol Transfus Cell Ther.

