# Supplementary material for: Transfusion transmitted infections among male blood donors of White Nile State, Sudan: Screening of the current seroprevalence and distribution
Source: BMC Res Notes. 2020 Nov 30;13:549. doi: 10.1186/s13104-020-05333-6 (PMC7708122; doi:10.1186/s13104-020-05333-6)
Supplement: Supplementary file 1 — Additional file 1: Figure S1. Rate of the serologic markers of multiple infections (MI) among TTIs positive cases. a overall MI. b and c show the rate of MI among age groups and localities, respectively. Pearson Chi -Squared test assessed the difference between groups. P value was < 0.001 (Age groups) and 0.088 (localities). [file 13104_2020_5333_MOESM1_ESM.docx]

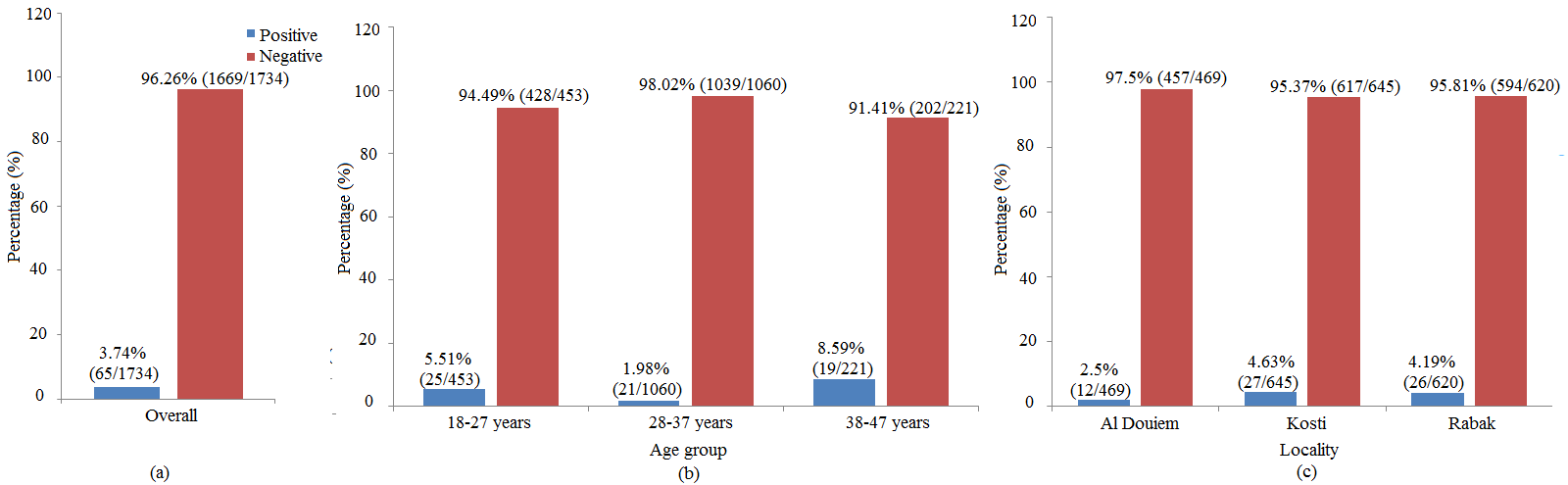


Figure S1. Probability of multiple of infections (MI) among TTIs positive cases. **a** overall MI. **b** and **c** show the rate of MI among age groups and localities, respectively. Pearson Chi -Squared test assessed the difference between groups. *P* value was ˂ 0.001 (Age groups) and 0.088 (localities).
